# Protocol for isolation of melanopsin and rhodopsin in the human eye using silent substitution

**DOI:** 10.1016/j.xpro.2023.102126

**Published:** 2023-03-07

**Authors:** Thomas W. Nugent, Drew D. Carter, Samir Uprety, Prakash Adhikari, Beatrix Feigl, Andrew J. Zele

**Affiliations:** 1 Centre for Vision and Eye Research, Queensland University of Technology (QUT), Brisbane, QLD 4059, Australia; 2 School of Optometry and Vision Science, Queensland University of Technology (QUT), Brisbane, QLD 4059, Australia; 3 School of Biomedical Sciences, Queensland University of Technology (QUT), Brisbane, QLD 4059, Australia; 4 Queensland Eye Institute, Brisbane, QLD 4101, Australia

**Keywords:** Neuroscience, Cognitive Neuroscience, Behavior

## Abstract

Melanopsin-mediated visual and non-visual functions are difficult to study *in vivo*. To isolate melanopsin responses, non-standard light stimulation instruments are required, with at least as many primaries as photoreceptor classes in the eye. In this protocol, we describe the physical light calibrations of the display instrumentation, control of stimulus artefacts, and correction of individual between-eye differences in human observers. The protocol achieves complete photoreceptor silent substitution in psychophysical, pupillometry, and electroretinographic experiments for probing melanopsin, rod, and cone function.

For complete details on the use and execution of this protocol, please refer to Uprety et al. (2022).^1^

## Highlights

• Steps to achieve independent control of photoreceptor excitations in the eye
• Modulate five photoreceptor excitations using five primary lights
• Instrument calibrations ensure photoreceptor isolation in a CIE Standard Observer
• Control for individual differences to ensure photoreceptor isolation in any observer

## Abstract (editor highlights)

Publisher’s note: Undertaking any experimental protocol requires adherence to local institutional guidelines for laboratory safety and ethics.

## Graphical abstract

**Figure:**
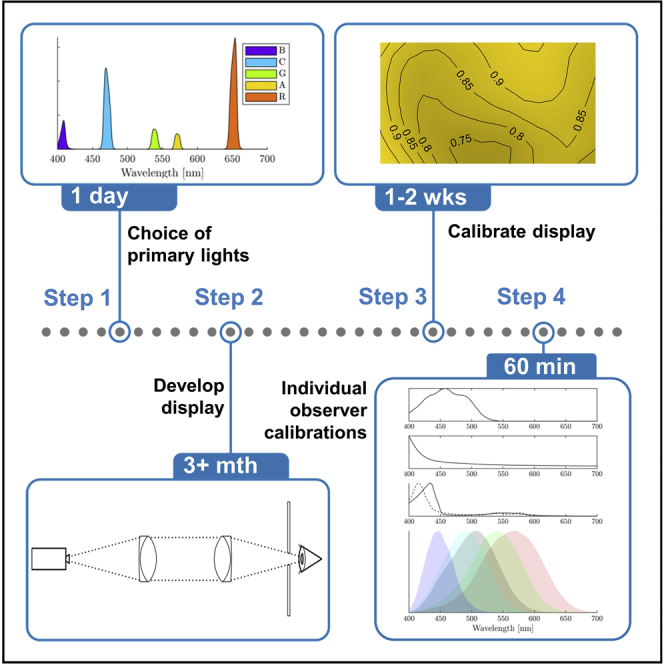

## Before you begin

### Performing silent substitution

This protocol describes the experimental procedures for conducting silent substitution^2^ across five photoreceptor classes. The silent-substitution procedure exploits the principle of univariance^3^ wherein the visual response to a stimulus is solely determined by the output response of each of the photoreceptor classes (determined with Equation 1). This means that spectrally distinct light sources which produce the same excitations across all photoreceptor classes will be indistinguishable to an observer. This principle holds where two light sources produce the same excitation across a subset of the photoreceptor classes (i.e., the silenced photoreceptor/s), such that any perceived difference will only be attributable to the photoreceptor class/es whose excitations changed between the two lights (i.e., the photoreceptor-directed stimulation).

**Figure 1:**
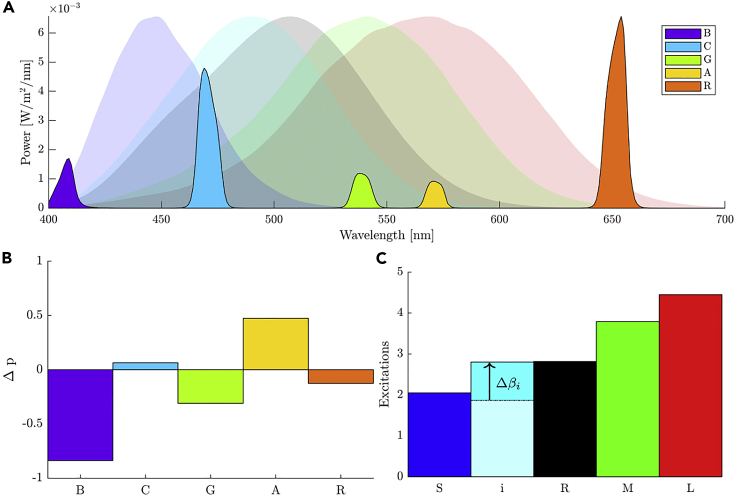
Photoreceptor-directed stimuli generated using the method of silent substitution (A) A set of spectral sensitivities of the five photoreceptor classes (S, M, L, R, i; shaded areas) are chosen according to step 1. The spectral outputs of five independently controllable narrowband primary lights (B, Blue; C, Cyan; G, Green; A, Amber; R, Red; saturated areas) are optimized according to step 2. (B) For the chosen photoreceptor spectral sensitivities and primary lights, the change in primary powers ($\Delta \vec{p}$) which produce a silent melanopsin-directed photoreceptor excitation change ($\Delta \vec{\beta }$) is determined according to step 3. (C) The change in melanopsin excitation ($\Delta \beta_{i}$) is shown with respect to a constant background adapting SMLRi excitation.

The five classes of photoreceptors in the human eye are the short (S)-, medium (M)- and long (L)-wavelength cones, rods (R) and melanopsin expressing intrinsically photosensitive retinal ganglion cells (i). Each class of photoreceptor preferentially absorbs light around a range of light wavelengths within the visible spectrum (Figure 1).

***Note:*** Non-visual opsins have been identified through gene expression and molecular studies of mammalian (including human) tissue; including an OPN3 encephalopsin,^4^ OPN5 neuropsin,^5^ and a retinal G protein-coupled receptor^6^ with a peak response towards the ultraviolet region of the spectrum. Because there is no direct evidence of a light-dependent role for any of these opsins in any human image-forming functions, these opsins are not considered in this silent-substitution protocol. Recordings in mice lacking rods, cones and melanopsin do not show any light-dependent changes in retinal ganglion cell firing, and so it remains to be determined whether light-dependent activation of other non-visual opsins can be communicated to the brain.^7^^,^^8^ If a role for these opsins were identified, the number and choice of primaries could be optimised for the silent substitution as per steps 1–4.

The relative likelihood that a photoreceptor class absorbs light at a certain wavelength is defined by the photoreceptor spectral sensitivity curves^9^^,^^10^ (Figure 1), which are available in the Toolbox for download at QUT Research Data Finder.^11^ As a result of these spectral sensitivity responses, the excitation $a_{i,j}$ of the i-th photoreceptor class by the j-th light/stimulus with a known spectral content can be found by cross-correlating the light’s spectrum at maximal power $P_{j}\left(\lambda \right)$ with each photoreceptor class’s spectral sensitivity response $s_{i}\left(\lambda \right)$:

(Equation 1) $$\begin{array}{c} a_{i,j}=\int P_{j}\left(\lambda \right)\;s_{i}\left(\lambda \right)d\lambda \end{array}$$

A photoreceptor-directed light is defined as a stimulus that changes the excitation of one, or a combination of specified photoreceptor classes whilst keeping the excitation of all other classes of photoreceptors constant (thus silent). For a display to generate a photoreceptor-directed light, it must have sufficient spectral robustness to independently control the excitation of all five photoreceptor classes. This protocol describes a method for producing photoreceptor-directed lights using a display which controls the spectral content of its output by mixing at least 5-primary lights whose output power is controlled. This mixture of 5 primary lights has a linear relationship with the photoreceptor excitations, defined by an A-matrix, with each element $a_{i,j}$ derived from Equation 1:

(Equation 2) $$\begin{array}{c} \vec{\beta }=\left[\begin{array}{ccccc} a_{S,B} & a_{S,C} & a_{S,G} & a_{S,A} & a_{S,R}\\ a_{M,B} & a_{M,C} & a_{M,G} & a_{M,A} & a_{M,R}\\ a_{L,B} & a_{L,C} & a_{L,G} & a_{L,A} & a_{L,R}\\ a_{R,B} & a_{R,C} & a_{R,G} & a_{R,A} & a_{R,R}\\ a_{i,B} & a_{i,C} & a_{i,G} & a_{i,A} & a_{i,R} \end{array}\right]\vec{p} \end{array}$$

where the proportion of output power of each of the five primary lights (compared with their maximum) is input as $\vec{p}={\left(B\;C\;G\;A\;R\right)}^{T}$ and the resulting photoreceptor excitations are output as $\vec{\beta }={\left(S\;M\;L\;R\;i\right)}^{T}$.

***Note:*** For the purpose of this protocol, we will label the 5 primaries as Blue (B), Cyan (C), Green (G), Amber (A) and Red (R), which tend to be descriptive of primaries that are often used.

#### Types of photoreceptor-directed stimuli for silent substitution protocols

A photoreceptor-directed stimulus light is specified for an experimental condition. With five photoreceptor classes in the eye, there can be five unique single photoreceptor-directed stimuli that modulate one opsin with the others remaining silent (e.g., melanopsin (i) -directed). Other photoreceptor-directed stimuli may target the post-receptoral pathways, including parvocellular (e.g., +L-M), koniocellular (e.g., S-cone) and magnocellular directed stimuli (e.g., +L+M luminance or +L+M+S achromatic). Photoreceptor interactions can be studied using combination stimuli (e.g., i and R stimuli with constant LMS excitation).

Before you begin the silent-substitution protocol, the following steps are required: (1) Choice of primary lights; (2) developing a display to present the photoreceptor directed stimuli; (3) calibration of the display; (4) performing individual observer calibrations; and (5) confirmation of silent substitution. Once these preliminary steps have been taken, an example experimental paradigm is presented to demonstrate an application of a silent substitution protocol.

### Institutional permissions

Prior to starting an experiment, institutional ethics approval must be obtained. Experiments are conducted in accordance with institutional ethics approvals and the laboratory health, safety and environment guidelines. Experiments involving humans are conducted in accordance with the tenets of the Declaration of Helsinki. The research purpose and study protocols are explained with written informed consent obtained from all participants.

### Choice of primary lights

**Timing: 1 day**

Any combination of at least five spectrally distinct lights can be used to perform silent substitution as described above; however, naïve combinations of primary lights will likely only be able to achieve a narrow range of single-photoreceptor modulations. This is due to the relationship between overlapping photoreceptor spectral sensitivity responses and the range of output powers of each of the primaries ($0\leq \vec{p}\leq 1$). Therefore, a preliminary analysis of the available sets of primary lights should be performed so that the chosen set of 5-primary lights is capable of the maximum achievable gamut of photoreceptor-directed stimuli.

1. Choose a set of spectral sensitivities for your experimental paradigm.

***Note:*** To perform silent substitution, it is necessary to have an estimate of the spectral responses of the different photopigment classes present in the eye of the test subject. The spectral sensitivity responses of the three cone photoreceptor classes that are derived from spectral measurements in dichromatic observers (having a reduced form of trichromatic vision) are termed König fundamentals. Such estimates include the Smith-Pokorny cone fundamentals^12^ which are transformations of the Judd-Vos corrected CIE 1931 colour matching functions, and the Stockman-Sharpe cone fundamentals which are transformations of the Stiles and Burch 10° adjusted to 2°.^13^

For experiments relating to melanopsin photoreception, the 10° diameter large field data (applicable for fields >4° diameter) are used in preference to the smaller 2° data as measurements of melanopsin function include the peripheral retina due to the ipRGC dendritic field being absent from and encircling the foveal pit.^14^ For different species, their relevant spectral responses can be implemented (Toolbox: *“Standard Observer (CIE)”*).^11^

2. Determine the spectral output of a set of available primaries.

***Note:*** A primary may be comprised of one or multiple illuminants; with or without spectral filters. To ensure the analysis can accurately evaluate the gamut of the primaries, the measured spectral output should be scaled to each primary’s maximum output (i.e., they should not be normalised). This can be done by measuring the spectral output of existing primaries, or from the manufacturer spectral data. This analysis may use a representative set of artificial Gaussian primaries (with a chosen full-width at half maximum (FWHM) bandwidth), but commercially available components might not adhere to those specifications.

***Note:*** The set of primaries used for this analysis should have a range of peak wavelengths across the visible spectrum (e.g., from 400 nm to 700 nm); the likelihood of the chosen primaries being close to optimal increases when more primaries are considered but the computational intensity of this analysis increases when the number of considered primaries goes from N→N+1 by a factor of $\left(\begin{array}{c} N\\ 5 \end{array}\right)/\left(\begin{array}{c} N+1\\ 5 \end{array}\right)=\left(N+1\right)/\left(N-4\right)$. The bandwidth of the primary choice will affect the analysis, where narrowband primaries will increase the gamut and maximum photoreceptor-directed contrast but will typically have a lower maximum radiance than wideband primaries.

3. Evaluate each set of 5 primaries. The chromaticity gamut of the system will be defined by the chromaticity coordinates of the 5 primaries that are chosen (Figure 5D).
  a. Generate a 5×N A-matrix for all N primaries considered using Equation 1.
  b. Loop over and extract each of the 5×5 A-matrices for the $\left(\begin{array}{c} N\\ 5 \end{array}\right)$ permutations of 5 primaries out of the N possible primaries.
  c. Compute the performance metric for each of the permutations of 5 primaries. The performance metric is informed by the intended experimental protocol.

#### Maximum photoreceptor-directed contrast (no pre-defined background chromaticity)

If the experimental protocol is intended to be conducted at any background adapting chromaticity, the maximum photoreceptor-directed contrast can be calculated as:

(Equation 3) $$\begin{array}{c} C_{Weber}=\frac{1}{-\sum_{j=1}^{5}a_{i,j}\;\min\left\{a^{j,i},0\right\}},\;C_{Michelson}=\frac{1}{\sum_{j=1}^{5}a_{i,j}\left|a^{j,i}\right|} \end{array}$$

where $a_{i,j}$ is element in the j-th row of the A-matrix corresponding to excitation of the i-th photoreceptor and $a^{i,j}$ is the element in the corresponding column and row of the inverse A-matrix.^15^ This metric will define the background adapting chromaticity of the intended experimental protocol, which may be unsuitable for the intended experiment.

#### Maximum photoreceptor-directed contrast at a pre-defined background chromaticity

If a certain background adapting chromaticity is required for an experimental protocol, for each permutation of 5 primaries considered the purely positive power, each of the three (of five) primary mixes which achieve the specified chromaticity should be calculated:

(Equation 4) $$\begin{array}{c} {\vec{p}}_{V}=B_{3\times 3}^{-1}{\left(x,y,z\right)}^{T},\forall {\vec{p}}_{V}\in R_{\geq 0} \end{array}$$

Where ${\left(x,y,z\right)}^{T}$ is the chosen background adapting chromaticity, $B_{3\times 3}$ is the matrix which relates the XYZ contribution of the three primaries to their output power^16^^,^^17^ and, ${\vec{p}}_{V}$ is the primary powers for the three primary mix which achieves the chosen chromaticity (where X+Y+Z=1). The powers of the 5-primary system after a maximum silent photoreceptor modulation from each of the three primary mixes (${\vec{p}}_{\mathrm{mod}}$) can be found with:

(Equation 5) $$\begin{array}{c} {\vec{p}}_{\mathrm{mod}}={\vec{p}}_{V}+K_{1}\;\Delta \vec{p},\;K_{1}=\min\left\{-\frac{p_{V,i}}{\Delta p_{i}}|\Delta p_{i}<0\right\}-\min\left\{\frac{p_{V,i}}{\Delta p_{i}}|\Delta p_{i}>0\right\} \end{array}$$

Where $\Delta \vec{p}$ is the change in primary powers which will silently modulate the target photoreceptor in the positive direction, found with $\Delta \vec{p}=A^{-1}\Delta \vec{\beta }$ when the change in photoreceptor excitation $\Delta \vec{\beta }$ is zero for all silent photoreceptors and one for the target photoreceptor. To calculate the scaling factor $K_{1}$, each negative or positive element in $\Delta \vec{p}$ (i.e., $\Delta p_{i}<0$ or $\Delta p_{i}>0$) should divide the corresponding element of ${\vec{p}}_{V}$ (i.e., $p_{V,i})$. The minimum functions will return a nonzero value if there is positive (the first minimum function) or negative (the second minimum function) modulation capacity from ${\vec{p}}_{V}$ in the silent $\Delta \vec{p}$ direction. This scaling factor can then be applied to $\Delta \vec{p}$ to find the maximum silent modulation from ${\vec{p}}_{V}$. From this the maximum contrast for a single, photoreceptor-directed stimuli at the chosen background adapting chromaticity can be given by:

(Equation 6) $$\begin{array}{c} C_{Weber}=\left|\frac{{\vec{\beta }}_{V}-{\vec{\beta }}_{\mathrm{mod}}}{\min\left\{{\vec{\beta }}_{V},{\vec{\beta }}_{\mathrm{mod}}\right\}}\right|,\;C_{Michelson}=\left|\frac{{\vec{\beta }}_{V}-{\vec{\beta }}_{\mathrm{mod}}}{{\vec{\beta }}_{V}+{\vec{\beta }}_{\mathrm{mod}}}\right| \end{array}$$

Where ${\vec{\beta }}_{V}=A{\vec{p}}_{V}$ is the photoreceptor excitation from the three-primary mixes and ${\vec{\beta }}_{\mathrm{mod}}=A{\vec{p}}_{\mathrm{mod}}$ is the photoreceptor excitation after the maximum, silent modulation (Toolbox: *“A matrix”*).^11^

***Note:*** When optimising for one of the above metrics, it is best practice to maximize the melanopsin and rhodopsin gamut given their lower contrast sensitivity than optimising for the cone pathways. This is because most primary choices will allow cone-directed stimuli with a contrast level above the contrast threshold of the cone pathways.

**CRITICAL:** Given that melanopsin and rhodopsin spectral sensitivities are largely overlapping, using four primary lights for a maximum contrast melanopsin- or rod-directed stimuli (ignoring rod or melanopsin excitation) will also produce a maximum contrast for the ignored rods/melanopsin. Rod-mediated visual responses are measurable in photopic illumination under conditions of silent substitution up to at least 8,000 Td (the maximum measurable illuminance with the instrumentation) which can lead to melanopsin-rod-cone interactions in photopic conditions.^1^

***Note:*** With more than 5 primaries, it is possible to obtain a broader gamut of background adapting chromaticities and larger photoreceptor-directed contrasts. These additional primaries can then be optimised to target multiple photoreceptors rather than individual photoreceptor classes. Five primaries are needed to make the A-matrix full rank, but more than 5 primaries will result in an overdetermined system, allowing for a broader range of solutions, even after non-negative constraints are considered. Despite this, the maximum achievable contrast for a photoreceptor-directed stimulus will be a 5-primary subset of those considered.

4. Select the optimal 5 primary combination.
  a. Limit the sets of 5 primaries considered to those which are the highest performing combinations of primaries based on the metric computed in step 3 (c).
  b. Evaluate the potential hardware errors from each of the sets of 5 primaries considered in step 4 (a) to see if the changes in primary powers during a photoreceptor-directed modulation are achievable with the intended hardware/modulator.
    Some $\Delta \vec{p}$ solutions may require large changes in some primaries and only small changes in other primaries. This may occur when one of the chosen primaries is centered at shorter or longer wavelengths. In this example case, the intended modulator may not have the bit depth resolution to smoothly change the primary output across a small number of levels (e.g., low contrast changes require higher bit depths). If a comparable performing set of 5 primaries exists for the performance metric which has a more even power distribution along the elements of $\Delta \vec{p}$, then this set of 5 primaries should be chosen as fewer hardware artefacts (i.e., open-field cone contrast errors; step 17) will be produced in the photoreceptor-directed stimuli due to bit level resolution.
    **CRITICAL:** The practical bit resolution achieved in a system is the number of output levels each primary can achieve between the two stimulus conditions. Where the vector requires a small change in an individual primary’s power between conditions, the bit resolution of the stimuli will be significantly lower than if the primary were to change over its entire power range. The practical bit-depth resolution is critical if the system is intended to measure threshold level vision. To counter this, the output power of the primaries can be scaled with an ND filter to increase the practical bit resolution.
    ***Note:*** When scaling the photoreceptor spectral responses, they can be normalized to peak, or scaled to some standard reference point. For example, the MacLeod and Boynton equiluminant cone chromaticity space^18^ or scaled by a spectral power distribution in the environment, such as daylight (e.g., CIE daylight D65).

### Developing a display to present the photoreceptor-directed stimuli

**Timing: 3+ months**

#### Instrumentation for stimulus generation

**Figure 2:**
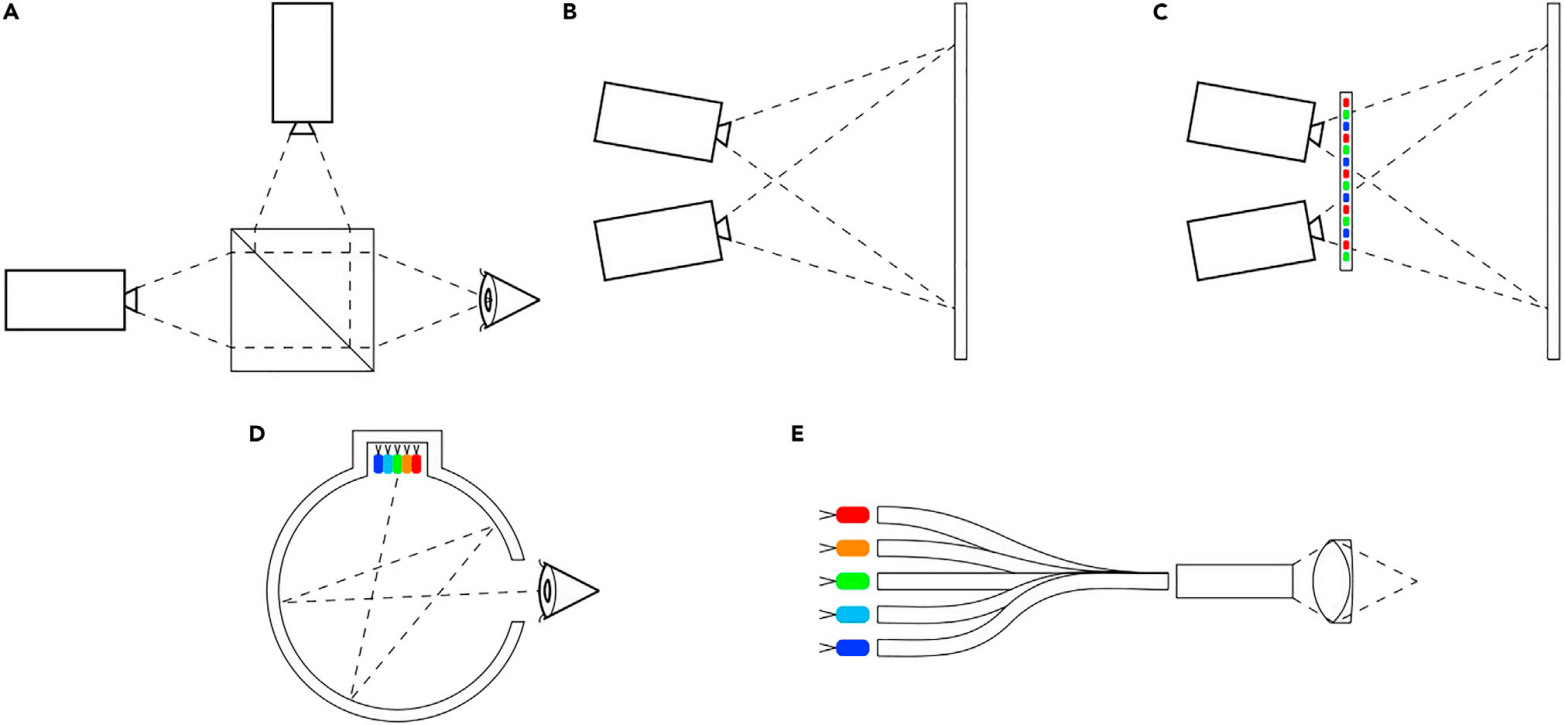
Instrumentation for stimulus generation There exist multiple solutions for generating spatial (A–C) and uniform (D and E) photoreceptor-directed stimuli. (A) Projectors which contain either one or a subset of the five primaries can be merged onto the same optical axis through the use of a beamsplitter. A simplified two projector system is shown here, where five projectors are merged in Nugent and Zele.^17^ (B) Two displays can be projected onto a screen, where each of the RGB outputs are filtered to create a five primary projection screen.^20^^,^^21^^,^^27^ (C) Two displays can be projected through an LCD where the projectors act as a backlight and the LCD modulates the pixel values.^24^ (D) A uniform five-primary system can be constructed by homogenizing their light outputs using a Ganzfeld integrating sphere,^26^^,^^19^ (E) or optical fibers and an integrating bar.^22^

**Figure 3:**
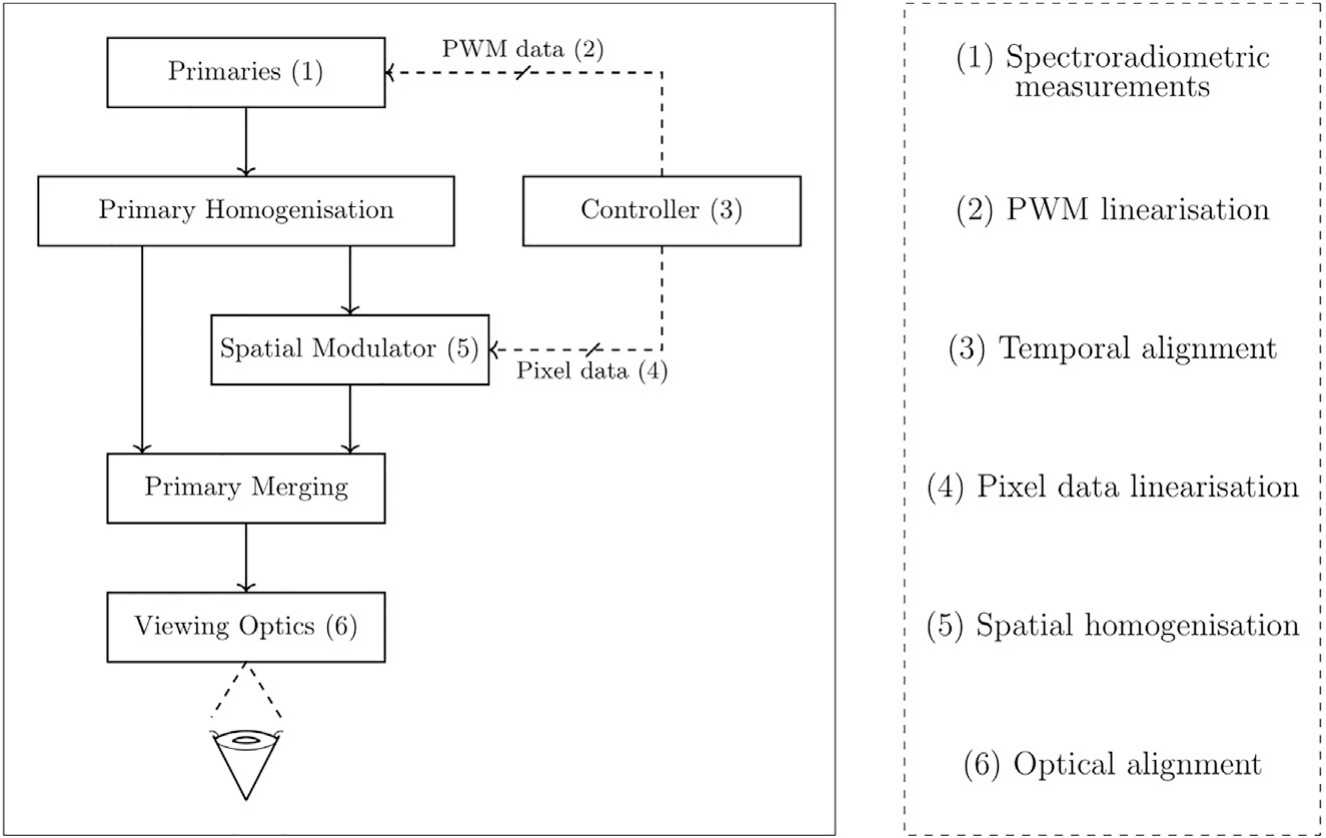
Developing a display to present the photoreceptor-directed stimuli Optical instruments and displays may differ in how they present visual stimuli, although typically the stimulus generator has some combination of primaries; homogenization; an illumination modulator; optics to present the stimuli to the eye; and a control method. Each of the typical calibration procedures have been labeled on the left panel with reference the system element which requires this calibration, as specified in the right panel.

This section of the protocol highlights the basic design principles and stimulus generators required for photoreceptor-directed silent substitution. Figure 2 shows exemplar systems used in this field.^17^^,^^19^^,^^20^^,^^21^^,^^22^^,^^23^^,^^24^^,^^25^^,^^26^^,^^27^ Because of the diversity of design options, this protocol lists the general considerations (Figure 3) that inform the design of a system rather than provide a step-by-step guide for a particular system (for a review of systems see: Conus and Geiser^28^; Nugent and Zele^17^).

The following steps guide the design principles of the stimulus generator:

5. Constrain the design properties of the visual stimulus generator by determining the type of images required to evaluate the experimental aims, namely:
  a. Uniform images (i.e., devoid of spatial details, except at the edges of the image)
    Systems which present purely temporal stimuli reduce the complexity of the device, including its control algorithm and calibrations. In addition, the reduced complexity of the stimulus brings the advantage that higher temporal frequencies can be probed (pulse width modulation (PWM) controller with many 1,000 s of oscillations per second).
  b. Spatially structured images (i.e., with spatial information)
    Systems designed to present spatially complex images will introduce a spatial modulator into the optical path. This could be a digital micromirror device (DMD) or liquid crystal display (LCD). The introduction of a spatial modulator requires a more complex control system to feed the spatial modulator with each image frame’s pixel information for each of the 5 primaries. Therefore, the maximum temporal frequencies achieved by such systems will be limited by the frame rate of the spatial modulator, which are typically much lower than the PWM limit of a light emitting diode (LED).
    ***Note:*** Stimulus devices capable of projecting uniform stimuli can only probe the temporal responses of the eye (with a fixed spatial configuration). Devices capable of projecting spatial images can probe both the spatial and temporal response properties of the visual system. Both devices can modulate the chromatic, achromatic and non-visual functions (e.g., pupil control pathway, circadian system).
    ***Note:*** Particularly in systems with higher LED current draw, the speed at which the LED reaches maximum intensity for a step input may be considered when determining optimal PWM or frame rates.
    6. Determine the mode of presentation view to the eye:
      a. Newtonian, or free viewing (i.e., uses the optics of the eye to focus the stimulus image onto the retina):
        Retinal illumination is dependent on the pupil size which is not controlled in Newtonian view systems, except with pharmacological pupil dilation, or inclusion of an artificial pupil (smaller than the smallest possible natural pupil). Pupil diameter can be influenced by age, disease and non-visual stimulations such as viewing distance, cognition, effortful or emotional responses.^29^ The retinal illuminations are also lower in Newtonian than Maxwellian view for the same light source because of pre-ocular light loss due to light divergence.
      b. Maxwellian view requires instrumentation designed so the exit pupil of the instrument is conjugate to the pupil of the eye.
        Maxwellian view affords control of the shape, size and focus of the stimulus independently of the pupil size to precisely control the retinal illumination.^28^^,^^30^^,^^31^^,^^32^^,^^33^^,^^34^ The position of the observer’s eye must be in the correct position during the entire experimental procedure.
    7. Homogenously distribute the primaries in the optical pathway that forms the retinal image.
    ***Note:*** In section 1, five primaries were chosen. The homogeneity of a retinal image is determined by the emission characteristics (source size and radiance) and transmission properties of the optical system. Light Emitting Diodes (LEDs) often used in modern visual stimulus generators do not emit uniformly at all angles and so require additional diffusion. The primaries can be homogenized, including from different sources, using diffusers, optical fibres, integrating bars^22^^,^^35^ or integrating spheres.^25^^,^^19^^,^^36^^,^^37^
    8. Select the modulating devices (uniform-field or spatial modulator) to generate stimuli from the primaries.
      a. Temporal modulator: Typically, a temporal modulator will be a controller (e.g., microcontroller or PWM chipset) which generates a PWM signal to the primaries. This PWM signal controls the output power of each primary. The switching speed (i.e., minimum duty cycle) of the controller will determine the bit depth resolution of the stimuli.
      b. (Optional) Spatial modulator: For a spatial display, the output stimuli are controlled on a pixel-by-pixel basis. The choice of spatial modulator (e.g., LCD or DMD) will determine the maximum spatial resolution, frame rate, and pixel level bit depth of the system.
    **CRITICAL:** For the bit-depth, temporal and spatial performance of the modulating device (i.e., frame rate and pixel density), continuous waveforms will be constructed as a series of step-changes that are the size of the minimum step size for these parameters (i.e., digitised). Consideration should be given to whether the presence of temporal and/or spatial artefacts from these step changes are visible to the eye. The minimum amplitude of the step changes is a function of the bit-depth. A low bit-depth may prevent measurement of threshold level vision; 8-bits is considered insufficient.
    A uniformly separated 8-bit system can represent 256 unique levels (including zero), which means that the minimum Weber contrast in a pedestal stimuli or Michelson contrast in a sinusoidal stimulus will be 1/254=0.39%. This contrast is above the minimum contrast visible to an observer under some of the most sensitive stimulus conditions; Pelli and Zhang^38^ give the example threshold contrast of 0.3% for a 3 c/deg sinusoidal grating but many other studies also report visual thresholds below 0.39%.^39^^,^^40^^,^^41^^,^^42^^,^^43^ In practice, a threshold experiment will typically titrate a participant’s contrast sensitivity starting from a suprathreshold contrast and passing through a subthreshold contrast. There exist strategies of obtaining lower contrast steps with an 8-bit system but these techniques either increase device complexity or sacrifice spatial or temporal resolution of the system.^38^^,^^40^^,^^44^^,^^45^

9. Determine how the stimulus will be generated. Commonly this involves control software and a communication protocol to interface between a computer running the experiment and the modulating devices. Examples include serial communication over USB to send PWM data to a microcontroller for temporal modulation, and HDMI video output (or other video protocol) into a DLP controller chipset for spatiotemporal modulation.

***Note:*** Hardware-based designs provide the lowest level timing control, but are typically constrained to the single, designed use case. For designs which require an operating system, Linux operating systems have the lowest-level software access, which allows the greatest control over OS-level interrupts. Operating systems such as Windows and MacOS are easier for researchers to use but may have OS-level interrupts that affect timing (despite processor power). High level languages often do not have sufficient low-level control of system timers to allow any research-level of timing determinism. One option to gain this level of control in MATLAB is PsychToolbox,^46^^,^^47^^,^^48^ which accesses low-level timing using GStreamer. Users should ensure the timing delays are shorter than the temporal response of the fastest physiological process under investigation.

10. Design the viewing optics and align the component elements to merge and homogenize the modulated stimuli and present it to the observer’s eye.

***Note:*** When designing the optical pathway, optical aberrations of the overall imaging system should be considered. Achromatic doublets can be used to reduce chromatic aberration primary lights of different wavelengths, especially at higher spatial frequencies.^49^^,^^50^

### Calibration of the display

**Timing: 1–2 weeks per display**

After completing the system design and construction, the photoreceptor-directed stimulus generator is calibrated to determine its performance characteristics and the reliability of the presentation of the study parameters with consideration to the following; (11) optical alignment; (12) photosensitive detectors for device calibration; (13) measurement units; (14) characterize the primary’s properties through the system; (15) spatial homogeneity of stimuli; (16) hardware timing and synchronization; measurement validation of the (17) open-field errors; (18) penumbral cone errors; and (19) perceptual validation.

11. **Optical alignment:** Align the optical components in the system. Perform this calibration step first because it may cause a later calibration step to become uncalibrated.
  a. Determine the level of alignment control that is needed from the system design. Uniform stimuli can be aligned at the primary homogenization step (i.e., in a Ganzfeld or integrating bar). Spatially modulated stimuli will require pixel level alignment across the entire stimuli where alignment may rely on control of all 6 degrees of freedom (e.g., x, y, z, pitch, roll and yaw).
  b. For a spatial modulator, present a structured image (e.g., a grid pattern) through the system and adjust each degree of freedom to reach pixel level alignment between projected images.

12. **Photosensitive detectors for device calibration:** Photosensitive detectors are used to sense the optical radiation for the physical light calibrations of the display instrument. Evaluate the available photosensitive detectors for applications in vision research in terms of the following:
  a. Spectral resolution: The detector resolution as a function of wavelength should have a high spectral resolution (e.g., 1 nm). Low spectral resolution detectors will result in an inaccurate A-matrix for the system because the error in the discrete integral increases as the spectral sampling width increases. This is particularly the case with narrowband primaries whose entire spectral response may be characterized within 10 nm.
  b. Sensitivity and noise floor: The bit depth resolution of the detector’s radiometric output is dependent on its sensitivity. The lowest output of the stimulus generator that is measurable by the detector will be determined by its noise floor. Higher sensitivity and a lower noise floor improve the signal-to-noise power ratio at low primary output levels. Many detector systems allow the signal-to-noise power ratio to increase by lengthening the integration time.
    ***Note:*** The noise source can be isolated by attenuating the primary output with a neutral density (ND) filter positioned in front of the detector. If the noise is attenuated, then the noise exists prior to the ND filter (e.g., within the system).
  c. Stability: The possible drift in the detector’s measurement of power outputs with time, temperature and/or exposure to optical radiation may require an evaluation of the detector’s warm-up characteristics and re-calibration of the dark light reference setting.
    ***Note:*** If required, the dark reference can be re-calibrated post-hoc by using the change in noise over time in spectral regions that do not contain any spectrum of interest (e.g., at the extremes of the primary outputs as a function of wavelength).
  d. Uniformity: The detector response may vary across its sensing area. This can be evaluated by moving the detector around a small-fixed aperture in front of a source light.

13. **Select measurement units:** The visible spectrum extends between about 400 nm to 700 nm. Common lighting metrics utilize 380 nm–780 nm, where this is more appropriate for human vision and other species extend to the UV/IR range. Light from a source location for vision experiments is characterized by its spectral power distribution. Some common categories of light measurement units are:
  a. Radiometric units independent of the eye, including radiance ($W\cdot m^{-2}\cdot sr$) and irradiance ($W\cdot m^{-2}$). These units are commonly used to describe the light source prior to entering the eye.
  b. Photometric units include luminance ($cd\cdot m^{-2}$) and illuminance (lx) for extended sources, and intensity (cd) for point sources. The units are commonly used to reference the light source to a function of a standard photometric observer (e.g., light source referenced to V(λ)) where that function is relevant to the interpretation of the stimuli (i.e., measuring cone pathway responses).
  c. Actinometric measures the quanta of photons which are incident at the eye ($\log\;quanta\cdot m^{-2}\cdot s$). These units can either be independent of the eye (corneal) or pre-processed to incorporate a standard observer’s pre-receptoral filtering (retinal). Actinometric units have a log-linear relationship with radiometric units and are useful to measure stimuli over a large range (e.g., Weber adaptive processes).

***Note:*** Retinal illuminance (Troland, Td) is the area of the natural or artificial pupil diameter multiplied by the source luminance. To convert photopic Td to scotopic Td, multiply by 2.49.^51^ An observer will experience a foveal scotoma when illumination levels are below cone threshold (∼1 photopic Td).

14. **Characterize the primaries’ properties through the system:** Integral to the methods of silent substitution is knowing the spectral power distributions and input-output relationship between the controller and the primary lights. When constructing stimuli from a combination of primary light outputs, the spectral content of the stimuli is the sum of the individual measured spectral content of each primary scaled by the chosen output power for that primary ($0\leq \vec{p}\leq 1$). Most, if not all systems will exhibit an amount of non-linearity between the desired and actual outputs. This relationship is defined by a look up table (LUT) in the form of linearization coefficients. The input-output linearization allows the user to control the average spectral composition of the stimulus.
  a. For calibration of a Newtonian view system, align the detector relative to the position of the eye of the observer. For Maxwellian view, align the detector with reference to the exit pupil of the device. Prior to completing the input-output measurements, warm-up the device, set the measurement output units, integration times, record the dark light, and if required, connect to a peripheral (e.g., computer), according to the detector instructions.
  b. Thermal stability: Use a photosensitive device to measure the maximum output and spectral content of the system over time. Take the first measurement when the device is first turned on and then periodically measure until the device reaches thermal stability.
    ***Note:*** Fluctuations in the primary light outputs due to thermal changes affect the precision of photoreceptor excitation calculations. If the device reaches thermal stability, the calibration measurements and test conditions should be performed after this onset period. If thermal stability is not reached, the heat-dependent spectral shifts and/or power reductions may be mitigated through the appropriate heatsinking or alternative light source drive currents.
  c. Spectral measurements: Characterize the spectral power distribution of each primary using a spectroradiometer. Measure each primary independently at maximum power (i.e., all other primaries switched off).
    ***Note:*** Depending on the technology used to modulate the primary, any variation in the spectral content over input levels (stable: LED and interference filter combinations with DMD or PWM, may change: CRT monitor, arc lamp with DMD; see: Barrionuevo et al.^31^) will introduce a chromaticity shift in the stimulus. Additional measurements across the input range should therefore be taken to confirm the static spectral content of the primaries.
    ***Note:*** The measured spectral outputs of each primary will have a noise floor across all measured wavelengths that are a property of the measuring device and not the primary. These would produce an erroneous contribution to the A-matrix (Equation 2). This can be corrected by identifying a range of significant measurements and removing spectral data outside of this range. The range of significant spectral data can be identified with the bandwidth of the primary (e.g., $\lambda_{\max}\pm 3\times Full\;Width\;at\;Half\;Maximum\;(FWHM$) or by setting a minimum threshold (i.e., noise floor amplitude) on either side of the peak, beyond which the value is set to zero (e.g., $1^{st}$ occurrence of 0.1% of maximum) (Toolbox: *“Trimmed LED Spectra”*).^11^
  d. Linearization measurements: To perform the input-output linearization, use a photosensitive device to measure the output power for each input level of each primary in the required measurement output units.
    ***Note:*** A photosensitive device which measures the integrated light across all wavelengths will typically have a higher signal-to-noise ratio in its measurements than a device which has spectrally separated measurements and therefore, can provide more accurate linearization measurements.
    ***Note:*** If illuminants/primaries are using a common power source, then take measurements to verify the output power of each primary is independent of the output power of all other primaries.
  e. Linearization modeling: Construct a look-up table (LUT) from the measured data to linearize the input-output relationship for each primary. Normalize the input ($in_{i}$) and output ($out_{i}$) values ($in_{i}=in_{i}/\max\left\{in_{i}\right\}$ and $out_{i}=out_{i}/\max\left\{{out}_{i}\right\}$) and then calculate a linearization coefficient for each measurement which will be the corresponding LUT value ($LUT_{i}$) with:
    (Equation 7) $$\begin{array}{c} {LUT}_{i}=\frac{{in}_{i}}{{out}_{i}} \end{array}$$
    Given a primary power solution ($\vec{p}$) to achieve a desired photoreceptor excitation ($\vec{\beta }$) (Equation 2), which was found through an A-matrix developed from spectral measurements at maximum power, then the actual primary power inputs required (${\vec{p}}_{lin}$) found through the LUT for the primary power solution with:
    (Equation 8) $$\begin{array}{c} {\vec{p}}_{lin}=LUT_{i}\times \vec{p} \end{array}$$
    If the input range is subsampled in the linearization measurements, then a mathematical model can be used to interpolate between the measured data points.

15. **Spatially homogenize the stimuli:** In any practical implementation, any primary will have a non-uniform power distribution across its projected area (i.e., hotspots) that require correction/compensation. When a stimulus is constructed from multiple primaries (i.e., 5-primary lights), the spatial distribution of power across the stimulus area will be different for each independent primary. Without correcting for the presence of inhomogeneities, the different spatial distributions of the spectral content of the primaries will result in a ratio of pixel-level photoreceptor excitations that vary non-uniformly across the stimulus area, thereby producing non-silent changes in the photoreceptor excitations.
  a. There are alternative ways to measure the spatial homogeneity of each primary in a stimulus depending on the available photosensitive devices and presentation view:
    i. Spot photometer: Project a nominally uniform field across the entire stimulus field. Focus and take a measurement with a spot photometer (e.g., PR655, BM-7) on each point in a grid pattern covering the stimuli field. This method is typically used in Newtonian systems, including with a rear or front projection screen, with a grid spacing that is large enough for the spot photometer receptive field to isolate localized regions.
    ii. Structured image projection: For a system with pixel-level control of the stimuli, divide the projected image into an equally sized regions and measure the output of each region when it is switched on individually. This method is useful for Maxwellian systems which are measured at the artificial pupil or exit pupil.
    iii. Camera: A high resolution camera can be used to measure spatial homogeneity. Cameras will have their own spatial response and a non-linear output relationship to input light levels. Both factors must be corrected to interpret the captured image as a spatial power distribution and image processing techniques to map pixel locations of the stimuli and the camera sensor.
  b. Interpolate the array of measurements to match the projected pixel output resolution so that individual pixels have a unique spatial weighting coefficient ($w_{i,j}$) for smooth transitions across the spatial weighting map.^17^ The center of a measurement region can be mapped to the center pixel from the range of pixels that measurement region represents.
  c. Create an inverse spatial homogeneity map from the interpolated spatial data ($m_{i,j}$) for each pixel value, where the pixel at location (i,j) can be scaled by a weighting coefficient ($w_{i,j}$) of:
    (Equation 9) $$\begin{array}{c} w_{i,j}=\frac{\max\left\{w\right\}}{m_{i,j}} \end{array}$$
    Where $m_{i,j}$ is the interpolated output of the pixel output at location (i,j). The weighting coefficient map should be rescaled by max⁡{w} so that the range of weightings across the map is between 0 and 1. This ensures that the spatial weighting map does not introduce upper limit clipping.

**CRITICAL:** Given that each pixel power is rescaled by the input-output linearization LUT and the spatial weighting coefficient, the practical bit-depth resolution of each pixel will be compressed. If this compression is significant, it may introduce non-silent pixel/regional artefacts. The spatial weighting will reduce the power output at the highest output pixel to be equal to the lowest output pixel. Potential solutions include better physical homogenization or higher bit-depth displays/controllers.

**Figure 4:**
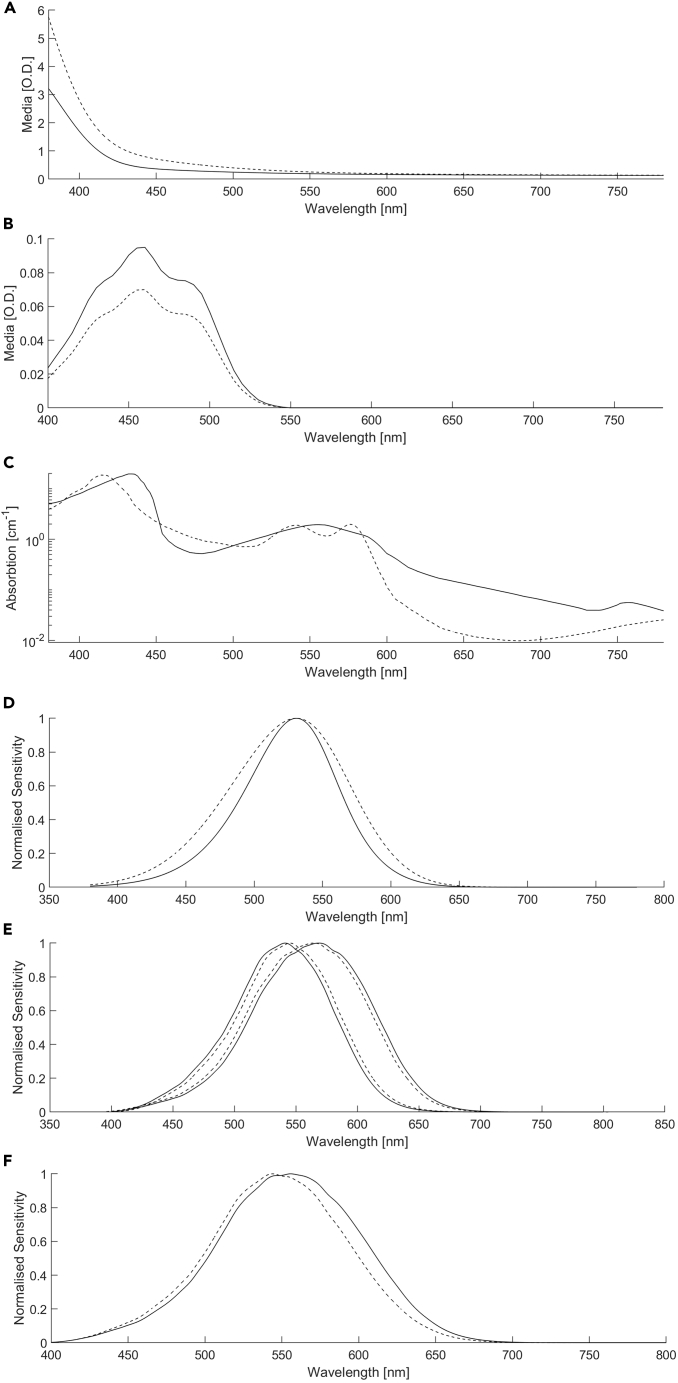
Sources of individual and experimental differences affecting the precision of the silent substitution Examples are presented for the 10° CIE standard observer (solid lines) and an exemplar individual observer (dashed lines). (A) Age-related increases in lenticular optical density manifest at shorter wavelengths and can vary across the lifespan by ∼25%. (B) The peak absorbance of the foveal macular pigment optical density (MPOD) at ∼460 nm can vary by ∼40%; its yellow pigmentation is associated with Maxwell’s spot. (C) The absorption of light by the hemoglobin and retinal vasculature attenuates the primary light spectrums such that open field photoreceptors and any photoreceptors located within the shadow of the blood vessels (i.e., penumbral cones) will receive a different light spectrum. The solid lines represent Hb and the dashed lines represent HbO2. (D) Photopigment optical density is illumination dependent. The example shown here is for an M-cone spectral sensitivity with a photoreceptor optical density of 0.7 (solid lines) and 0.1 (dashed lines). Corrections are applicable at higher adaptation levels (>8000 photopic Td). Typically, no change is required for the mean viewing illuminations achieved with standard visual displays. (E) The presence/absence of gene polymorphisms determine the L- and M-cone peak spectral separation. There are no known polymorphisms affecting the rhodopsin or S-cone opsin spectral response. There is no evidence that OPN4 polymorphisms alter the peak sensitivity or affect melanopsin dependent visual responses. There may be some evidence that OPN4 polymorphisms produce a functional change of melanopsin photoreception through a proxy of sleep onset and the PLR constriction amplitude but measurements of the melanopsin-mediated PIPR amplitude do not show any functional change. (F) The luminous efficiency of an individual is also dependent on their L:M cone ratios, which for a standard observer is ∼2:1 and in individuals can vary between ∼0.5:1–∼15:1.
  Estimate the penumbral cone contrast as follows:
  a. Measure the spectroradiometric output of the display at the extreme ranges of the photoreceptor-directed stimulus (i.e., measure the melanopsin-low and melanopsin-high conditions).
  b. Filter the measured spectroradiometric outputs with the spectral absorption of hemoglobin.^60^ Using the filtered spectra, calculate the penumbral cone [S,M,L] photoreceptor excitations.
  c. Calculate the penumbral cone contrast present from the differences in penumbral cone excitation between the extreme ranges of the photoreceptor-directed stimulus. To mitigate the penumbral cone errors, refer to the observer calibration procedures described in step 20.
    ***Note:*** The absorption $\alpha_{total}\left(\lambda \right)$ of the primary light power by haemoglobin^60^ is given as:
    (Equation 10) $$\begin{array}{c} \alpha_{total}\left(\lambda \right)=0.85\alpha_{HbO_{2}}\left(\lambda \right)+0.15\alpha_{Hb}\left(\lambda \right) \end{array}$$
    where the absorbance coefficients of oxyhaemoglobin $\alpha_{HbO_{2}}\left(\lambda \right)$ and deoxyhaemoglobin $\alpha_{Hb}\left(\lambda \right)$ are defined as 5×ln(10)×0.002326×ε(λ)/1000, where ε(λ) is the molar extinction coefficient, 5 μ m is the average central retinal capillary diameter within 3.5 mm eccentricity (∼23.4° field of view),^61^ 0.002326 is the hemoglobin molar concentration defined by the ratio of the hemoglobin concentration (150 gm/litre) to gram molecular weight (64,500).^62^
    The combined HbO2 and Hb absorbance is then $0.85\alpha_{HbO_{2}}\left(\lambda \right)+{0.15\alpha }_{Hb}\left(\lambda \right)$ based on the average of the HbO2 proportions in arteries (95%) and veins (75%).^63^ The absorbance is wavelength dependent with the peak at shorter wavelengths between 418 and 436 nm and therefore differentially affects the 5 primary lights’ spectral radiances received by penumbral photoreceptors.

16. **Synchronize hardware timing:** The complexity of the stimulus design and temporal synchronization of the primaries depend on the test protocol. In general, the minimum sample rate of a system will depend on the visual processes under investigation; for example, the melanopsin pathway has low temporal sensitivity (<5 Hz), the rod pathway a faster response (<30 Hz), and the cone pathway is sensitive to frequencies approaching 100 Hz. The temporal response of cells in the visual pathways are higher in the retina than cortex.^52^ The highest possible sample rate should be used for the stimuli and the measurement peripherals recording the photoreceptor directed responses of interest (e.g., measures of the pupil control pathway, electroretinogram).
  As a general approach that will ensure temporal synchrony across most system designs, use a photosensitive diode and a data acquisition system (i.e., oscilloscope) to evaluate the timing and synchronization of the temporal onset of primary frames:
  a. Position a photosensitive diode as required for measurement in the chosen presentation view.
  b. Generate a maximum power stimulus (with no spatial weighting applied) which produces a step change in output across all primaries so that the duty cycle of any PWM is 100%.
  c. Measure the frame onset across all primaries. Without any temporal delay between primaries the theoretical change in output levels will be a single step change in output. An inter-primary temporal delay will appear as multiple, time-delayed, step changes. The relative differences between these delayed step changes are defined as the inter-primary temporal delay. If the controller of the system is non-deterministic the inter-primary temporal delays will not be constant and can instead be expressed as summary statistics of the largest inter-primary delays over frames.
  d. Model the non-silent artefacts arising from the measured temporal delays as a function of photoreceptor excitations across the intended stimulus protocol.
    ***Note:*** For high frame rate systems the energy of these artefacts will be lower and so may be below the temporal resolution of the eye. Any significant temporal artefacts can be mitigated by using smoothly transitioning waveforms and raised-cosine envelopes for stimulus windowing.
    ***Note:*** The source of temporal artefacts could originate in the hardware design of the system or the software of the system/protocol. These may require consideration be given to effective memory and communication management. Software-based buffering and code prioritisation is also a possible solution to synchronisation issues. For PWM or custom hardware the temporal delays between components can be measured by an oscilloscope probe.
  e. Peripherals: In addition to measuring the inter-primary delays, also quantify the relative timing difference between frame onset and the timing onset of peripheral recording devices (e.g., cameras, response buttons, acquisition devices for electrophysiological recordings).
17. **Measurement validation - Open-field errors:** The system’s ability to generate a photoreceptor-directed stimulus can be validated by presenting a stimulus with a calculated set of photoreceptor excitations and using a spectroradiometer to measure its spectral content. The actual photoreceptor excitations from measured spectral data will determine the precision of the photoreceptor-directed stimulus for the standard observer, ensuring the measured and calculated excitations match. Any differences between the measured and computed excitations will produce a (physical) open field cone contrast artefact. This is sometimes called splatter.^53^^,^^54^
  The implication of open-field contrast is that the photoreceptor directed stimulus may not be completely silent because some signal contrast is unintentionally presented to the theoretically unmodulated photoreceptor classes. Whether or not this artefact affects the silent substitution will depend on the magnitude of the artefact and the stimulus design. The open field contrast can be estimated as follows:
  a. Measure the spectroradiometric output of the display at the extreme ranges of the photoreceptor-directed stimulus (i.e., measure the melanopsin-low and melanopsin-high conditions) and calculate the measured [S,M,L,R,i] photoreceptor excitations.
  b. Calculate the theoretical [S,M,L,R,i] photoreceptor excitations by replacing α in Equation 2 with these measured output values.
  c. Find the open-field cone intrusion in the photoreceptor-directed stimulus as the difference between the measured (step a) and theoretical (step b) [S,M,L,R,i] photoreceptor excitations quantified/calculated using the standard observer functions.
  d. Report the (physical) open-field L-, M- and S-cone and rhodopsin contrasts for the melanopsin-directed stimulus, and vice versa for rhodopsin- or cone-directed stimuli.^55^
  e. Determine the perceptibility of this open-field contrast by presenting the measured open-field contrast (without the intended photoreceptor-directed excitation change) to an observer.^55^ If the system does not have the resolution to present the exact open-field contrast, present the next highest (and achievable) contrast for this test.
    ***Note:*** The level of acceptable open-field errors is dependent on the most sensitive visual process under the viewing conditions (e.g., for the stimulus eccentricity, spatio-temporal, illumination); this will most-likely be a cone-mediated chromatic or achromatic pathway response.
    **CRITICAL:** To avoid rod photoreceptor interactions that can influence melanopsin-directed visual contrast sensitivity, melanopsin-directed stimuli should have less than 3% rod contrast.^1^

18. **Measurement validation - Penumbral cone errors:** The photoreceptor-directed stimulus is designed based on the spectral sensitivities of the open field photoreceptors (step 1). Due to the absorption of light by the retinal vasculature, any photoreceptor located within its shadow (i.e., penumbral cones) will receive a different light spectrum to the open field photoreceptors (Figure 4C). The result of this for a photoreceptor-directed stimulus is that penumbral cone photoreceptors are not silenced.^56^^,^^57^^,^^58^^,^^59^

19. **Perceptual Validation:** What does a photoreceptor-directed stimulus look like? On completion of the display calibration, use the visual appearance of a cone-directed stimulus to provide a preliminary evaluation of the system calibrations and software computations.
  a. Generate cone-directed stimuli (e.g., +L-M, S-cone directed, +L+M+S) using the A-matrix with the standard observer functions.
    ***Note:*** It is preferable to conduct the temporal perceptual tests using uniform stimulus patches with a square edge (e.g., 1 s duration) presented on a neutral white adapting field to minimize inadvertent effects of chromatic and achromatic surround contrast on the visual appearance of the photoreceptor directed stimuli.
  b. Perceptually evaluate the visual appearance of suprathreshold cone-directed stimuli on a white adapting background level, where the untargeted photoreceptors are silent:
    i. Chromatic modulation (+L-M): Stimulus increments from the background will appear magenta and decrements will appear cyan.
    ii. Chromatic modulation (+S): Stimulus increments from the background will appear purple and decrements will appear lime.
    iii. Achromatic modulation (+L+M+S): Stimuli increments will appear as a luminance increase without a change in chromaticity (roughly speaking, appear brighter) and decrements will appear dimmer without a chromatic change.
      ***Note:*** The spectral signatures of the cone-directed opponent neuronal responses do not directly match the phenomenological experience (i.e., a magenta-cyan appearance for the putative red-green colour direction) due to the multistage processing of these photoreceptor directed input signals.^64^^,^^65^

Once the system calibrations have been performed and initially confirmed with a preliminary assessment; complete the individual observer calibration prior to conducting the silent-substitution experiment.

### Performing individual observer calibrations

**Timing: 30–60 min per participant per step**

An observer participating in a silent substitution experiment may differ in some way from the standard observer functions,^10^ as defined by the International Commission on Illumination^9^ (Toolbox: *“Standard Observer (CIE)”*).^11^ Regardless of the cone fundamentals chosen, the individual differences between observers will be greater than the differences between the cone fundamentals. As shown in Figure 4, individual differences occur due to some combination of variations in macular pigment optical density (MPOD), lenticular optical density, gene polymorphisms and photopigment spectral responses, and photopigment optical density.

These individual differences result in larger A-matrix errors when using visual stimulus generators with narrowband primaries compared to broadband primary systems (though the latter will have a smaller gamut).^66^ These individual differences can be accounted for in the A-matrix calculation by the application of an individual observer calibration. An individual observer calibration will result in each column of the A-matrix being rescaled to account for the change between the actual and standard observer’s photoreceptors (see step 23 for a description of this process).

***Note:*** Observer calibrations should be performed in the same retinal location as for the silent substitution experiment. Individual differences in macular pigment filtering can be ignored if the center (e.g., ∼ $10^{\circ }$ diameter) of the stimulus field is occluded, or by using eccentric retinal fixation to position the stimulus field outside the macular pigment. If the stimulus is projected into the central retina, an entopic phenomenon known as Maxwell’s spot may occur due to the absorption of short wavelength light by the macular pigment; this depends on the primary choices.

**Figure 5:**
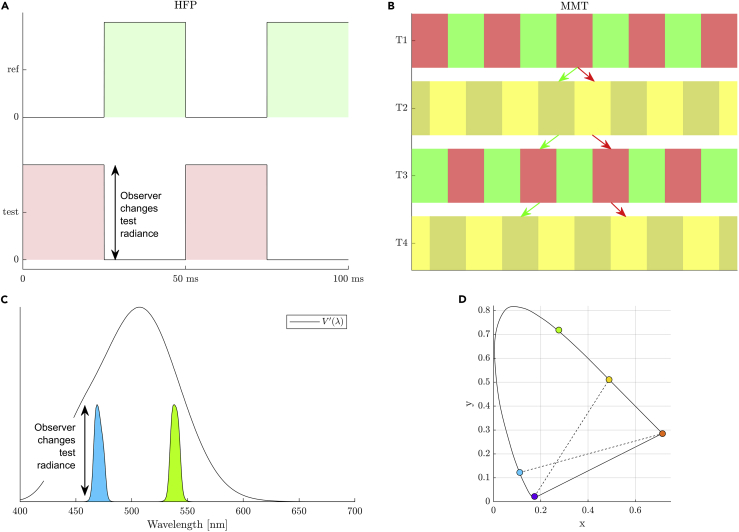
Performing individual observer calibrations One of the following procedures should be performed. (A) Following step 21(a), the heterochromatic flicker photometry (HFP) procedure includes a reference primary (e.g., green) that is temporally alternated in counterphase with a test primary (e.g., red). The observer is tasked with changing the radiance of the test primary to minimize or eliminate the perception of flicker. At the criterion setting, the ratio of the test and reference primary amplitude is recorded ($k_{p}$) and used to scale the individual observers A′ matrix (step 23). (B) Following step 21(b), the minimum motion technique (MMT) requires an observer to minimize the induced motion from a spatially structured square wave grating which temporally alternates between a grating of two primaries (T1 and T3; e.g., a green reference and red test primary) and a grating of a light and dark mixture of the two primaries (T2 and T4), by controlling the radiance of the test primary in the odd time intervals. Induced leftward motion is perceived (green arrow) when the test primary has a higher luminance contribution than the reference primary. Induced rightward motion is perceived when the test primary has a lower luminance (red arrow). At the criterion setting, the ratio of the test and reference primary amplitude is recorded ($k_{p}$) and used to scale the individual observers A′ matrix (step 23). (C) Following step 21(c), a scotopic color match is made between the reference primary (e.g., green) and another test primary (e.g., cyan) that has a variable radiance (excluding the long wavelength primary). The test-to-reference ratios at the isomeric matches are used to scale the rhodopsin (R) row of the individual A-matrix (Equation 13) for the four measured primaries (excluding red). A photopic color match (right panel) is then made between four (of five) primaries, including the long wavelength (red) primary as shown in (D) the 1964 CIE chromaticity diagram. Together the scotopic and photopic color matches correct the individual observer’s pre-receptoral filtering of the five primaries (step 23).
  b. **Minimum Motion Technique (MMT):** For structured stimuli, the minimum motion technique^79^ can be used to estimate an individual’s differences in pre-receptoral filtering and spectral sensitivities of the L- and M-cones. This technique minimizes the induced motion from a square wave grating which alternates between a grating of two primaries and a grating of a light and dark mixture of the two primaries. This method is performed as follows (Figure 5B):
    i. Generate a spatial, square wave grating which changes across four time increments, where the spatial grating shifts in phase by π/4 radians at each time increment.
      ***Note:*** At odd time increments the spatial, square wave grating will alternate between a reference (e.g., green) and test primary. At even time increments the spatial, square wave grating alternates between a light (high luminance) and dark (low luminance) mixture of the reference and test primary, where this mixture is an equal chromatic mixture of the two primaries.
    ii. Have the participant control the output radiance of the test primary in the odd time increments. When the test primary at a chosen radiance has a higher luminance contribution than the reference primary, an induced leftward motion is perceived by the observer. When the test primary has a lower luminance, an induced rightward motion is perceived.
    iii. The participant is tasked with minimizing the apparent motion of the stimulus. The point of minimum perceived motion will be the isoluminant point for the reference and test primary.
    iv. Record the ratio of test primary radiances compared to the reference primary for the points of minimum apparent motion as a set of ratios, ${\vec{k}}_{p}={\left(p_{B}/p_{G},p_{C}/p_{G},1,p_{A}/p_{G},p_{R}/p_{G}\right)}^{T}$.
    v. Use the ratios to scale each test primary relative to that of the reference primary ($k_{p}$) in the individual observer A’ matrix (Equation 14 in step 23).
      ***Note:*** MMT results are dependent on spatial but not temporal frequency.^79^ Since this method relies on the subjective perception of motion direction, it can also be used in children^80^ and animals^81^ by predicting the perceived motion through optokinetic nystagmus.
  c. **Scotopic color matches.** Because there are no rhodopsin polymorphisms,^82^ any deviation in an individual’s scotopic color match from that predicted by the standard observer rhodopsin nomogram (scotopic V’(λ)) will represent the individual’s pre-receptoral filtering. This method^35^^,^^83^ quantifies the lens attenuation and therefore can scale all photoreceptors (though it does not account for L:M cone ratios). The color matching technique is as follows (Figures 5C and 5D):
    i. At a scotopic light level, present the reference (e.g., green) and test primary in a bifurcated field. This step will not measure long wavelength primaries (i.e., >620 nm) because the photochromatic interval for rods and cones will approach zero.
    ii. The radiance of the reference primary is fixed, and the observer changes the radiance of the test primary until the rod pathway perceives the two primaries as having equal brightness. Record the individual observer’s test-to-reference radiance ratio ($r^{ind}$) at the isomeric match.
    iii. For the standard observer, find the test-to-reference radiance ratio ($r^{std}$) for the j-th primary through the A-matrix:
      (Equation 12) $$\begin{array}{c} r_{j}^{std}=\frac{a_{R,j}}{a_{R,ref}} \end{array}$$
      Where $a_{R,j}$ is the element of the A-matrix corresponding to the rhodopsin (R) row and the j-th primary column, and $a_{R,ref}$ corresponds to the reference primary column (e.g., green). This means that the per-primary scaling coefficients to account for the pre-receptoral filtering is found with:
      (Equation 13) $$\begin{array}{c} k_{p}=\frac{r^{ind}}{r^{std}} \end{array}$$
    iv. To estimate the individual observer’s pre-receptoral filtering of the long wavelength primaries (e.g., red), perform a photopic colorimetric match between four (of five) primaries, including the long wavelength primary.
      Complete the colorimetric match by equating the tristimulus values of the mixture of a pair of primaries (e.g., B+G=C+R), where the radiance of the three reference primaries (e.g., not red) are held constant and the radiance of the test primary (e.g., red) is changed by the observer.
      The initial condition of this photopic match begins with ratios of primary radiances which are a match for the standard observer. The per-primary scaling coefficients from the scotopic match are used to rescale this initial condition, except for the test primary (which does not yet have a scaling coefficient).
    v. Have the observer alter the radiance of the test primary to obtain a satisfactory colorimetric match. The scaling coefficient for this test primary will be the ratio of the radiance required for a match between the individual and standard observer.
    vi. Use the ratios to scale each test primary relative to that of the reference primary ($k_{p}$) in the individualized observer A’ matrix (Equation 14 in step 23).
22. (Optional) Alternate methods to account for individual observer differences. The methods listed above scale the power outputs of each primary to account of individual observer differences in the A-matrix. The following methods perform the same individual observer calibration through different means:
  a. **Cone metamers:** With structured spatial stimulus patterns, empirically defined metamers can produce theoretically metameric stimuli for the cones by accounting for individual variances in cone spectral sensitivity and/or pre-receptoral filtering.^84^ The following describes how cone metamers can be determined:
    i. Choose a desired adapting chromaticity (e.g., x,y,z=[1/3,1/3,1/3]) and determine the primary powers required to produce the minimum and maximum melanopsin stimuli using the metric outlined in step 3. Use the minimum melanopsin excitation as the reference stimulus and the maximum melanopsin excitation as the test stimulus (i.e., the test stimulus begins as the standard observer cone metamer).
    ii. For an individual observer the initial test stimulus may not be a cone metamer due to individual differences. The true individual observer’s cone metamer should exist close to the standard observer’s cone metamer. Following this assumption, generate a set of potential cone metamer matches by allowing the SML excitations to change by less than some threshold (i.e., <1%) from the standard observer cone metamer. These permutations form the set of test stimuli.
    iii. Present stationary, sinusoidal, spatial gratings where the reference and test sinusoid (i.e., the potential cone metamer) are presented counterphase to each other. A spatial frequency is chosen to which the cones have high spatial contrast sensitivity (e.g., 3.2 cycles/°); the grating is presented to the peripheral retina.
    iv. The grating can have one of four orientations (i.e., 4 alternative spatial forced choice procedure) and the observer is tasked with correctly identifying the grating orientation.
    v. An individual observer’s cone metamer (corrected for individual observer differences) is any potential cone metamer such that the observer cannot correctly identify the grating orientation.
    vi. The inference is that a modulation between the reference (melanopsin low) and individual observer’s cone metamer (melanopsin high) will continue to be a cone metamer.

***Note:*** Many spectral composition samples need to be examined to counter the possible range of individual observer differences. The reference and test stimuli are presumably silent for the cones, but there is no reason why this procedure won’t silence melanopsin. Although this technique requires the working assumption that neither melanopsin nor rods contribute a visual percept in the initial viewing conditions (i.e., the low melanopsin and rhodopsin excitation), both the rod^85^^,^^86^ and melanopsin pathways do mediate colour^53^^,^^55^^,^^67^^,^^87^ and brightness percepts.^36^^,^^88^^,^^89^^,^^90^^,^^91^

20. Choose a stimulus protocol which will suppress penumbral-cone errors. This class of techniques suppress the output of the intended silent photoreceptors (e.g., cones, including from penumbral cones) and are often sufficient in reducing individual observer differences to below threshold. One of the following two techniques should be incorporated into the silent substitution protocol.
  a. **Temporal white noise (TWN).** Visual perception is initiated by the most sensitive processes. Penumbral-cone artefacts in a melanopsin-directed stimulus will inadvertently mediate vision at higher temporal frequencies beyond the melanopsin critical flicker frequency (CFF).^1^^,^^67^ To eliminate these intrusions:
    i. Generate temporal white noise.^67^^,^^68^ Create this TWN as a sum of temporal sinusoidal signals which differ across all achievable frequencies for the system and have a uniformly distributed phase offset between each frequency, namely:
      (Equation 11) $$\begin{array}{c} TWN\left(t\right)=\sum_{n=1}^{N}\cos\left(2\pi nf_{0}t+\varphi_{n}\right),\;\varphi_{n}∼\;U[0,2\pi ) \end{array}$$
      Where ${Nf}_{0}$ is the highest temporal frequency which is achievable by the system and $f_{0}=1/T$ is the lowest frequency component of the TWN determined by the reciprocal of the TWN duration. If the TWN noise is generated so that it targets the intended silent photoreceptors (e.g., SMLR noise and silent melanopsin) then the amplitude of the TWN can be multiplied by a photoreceptor-excitation as desired (i.e., the primary powers which modulate SMLR but not melanopsin) (Toolbox: *“Stimulus”*).^11^
    ii. Present the TWN to the observer during the pre- and post- stimulus interval. For example, a TWN presentation of 1 s duration during the pre- and post-stimulus intervals will desensitize penumbral cones for ∼1 s following noise offset.^67^
      (Optional) If you have a display capable of generating structured images, you can generate spatiotemporal white noise across each pixel with Equation 11.
      ***Note:*** Temporal white noise based on the standard observer functions (i.e., without individual observer calibrations) is sufficient to desensitize penumbral cone intrusion and minimize the effect of any individual observer differences for melanopsin-directed stimuli.^69^
      **CRITICAL:** If the TWN is applied with reference to the standard observer functions, there may be an error in estimated retinal illumination of the adapting chromaticities if the individual and standard observer luminous efficiencies do not match.^69^ This is important for measurement at the transition between scotopic-mesopic-photopic illuminations. This error can be corrected by applying observer calibrations.
  b. **Low temporal frequency stimuli:** If TWN is not applied, restrict the temporal frequency of all stimuli to ≤1 Hz so as to avoid penumbral-cone errors.^58^ The individual observer calibrations which do not suppress penumbral cone intrusion are sufficient if these low temporal frequency test stimuli are used.
21. Perform an individual observer calibration for each participant to minimize the differences between the standard observer and that individual observer.^70^ One of the following calibrations should be performed to rescale the standard observer A-matrix:
  a. **Heterochromatic Flicker Photometry (HFP):** Differences that arise from variations in pre-receptoral filtering and spectral sensitivities of the L- and M-cones can be estimated with HFP.^71^ Heterochromatic flicker photometry provides an estimate of an individual’s luminosity function V(λ).^69^^,^^72^ The HFP method is as follows (Figure 5A):
    i. Specify the primary that produces the most similar quantal catches for the L- and M-cones as the reference primary (e.g., green).
    ii. Temporally modulate the reference primary in counterphase with one of the other test primaries (randomized: Blue, B; Cyan, C; Amber, A or Red, R). For flicker perception to be mediated via the luminance pathway, the presented temporal frequency (e.g., ≥ 15 Hz) should be beyond the resolution of the chromatic pathways.^73^^,^^74^
    iii. Have the observer change the amplitude of the test primary to minimize or eliminate the perception of flicker. The amplitude of the reference (green) is kept constant.
    iv. At the criterion of minimal flicker the ratio of the test and reference primary amplitude is recorded as ${\vec{k}}_{p}={\left(p_{B}/p_{G},p_{C}/p_{G},1,p_{A}/p_{G},p_{R}/p_{G}\right)}^{T}$.
    v. Use the ratios to scale each test primary relative to that of the reference primary ($k_{p}$) in the individual observer A’ matrix (Equation 14 in step 23).
      ***Note:*** The mean retinal illuminance should be approximately 50 photopic Td (124 scotopic Td) to satisfy Abney’s law of additivity.^75^^,^^76^ The adapting chromaticity is chosen to minimize L- and M-cone adaptation (e.g., EEW or Neutral white).^13^^,^^77^ The minimally distinct border (MDB) method^78^ also yields spectral sensitivity functions close to that obtained by HFP.

**CRITICAL:** The metameric match may be valid only for a limited range of spatio-temporal variations in the stimulus composition from the initial matching conditions. Verify the limits to which the metamers hold with changes in the exposure duration, after images, adaptation, habituation, and control for the Troxler effect during peripheral fixation (i.e., fading).

**CRITICAL:** Ensure the bit-depth of the primaries has sufficient resolution to titrate the potential cone metamer threshold.

In summary, we recommend the HFP and color matches observer calibration methods for uniform displays and the MMT and cone metamers methods for structured displays including gratings and checkerboards as it can be performed with broad spatial and temporal frequencies. Scotopic color matches can be used to quantify the observer pre-receptoral filtering.

23. **Individual observer A′ matrix:** The primary amplitude ratios from the observer calibration (${\vec{k}}_{p}$) are used to scale each primary relative to that of the reference primary in the individual observer’s A’ matrix, where:
  (Equation 14) $$\begin{array}{c} \vec{\beta }={\vec{k}}_{p}\left[\begin{array}{ccccc} a_{S,B} & a_{S,C} & a_{S,G} & a_{S,A} & a_{S,R}\\ a_{M,B} & a_{M,C} & a_{M,G} & a_{M,A} & a_{M,R}\\ a_{L,B} & a_{L,C} & a_{L,G} & a_{L,A} & a_{L,R}\\ a_{R,B} & a_{R,C} & a_{R,G} & a_{R,A} & a_{R,R}\\ a_{i,B} & a_{i,C} & a_{i,G} & a_{i,A} & a_{i,R} \end{array}\right]\vec{p} \end{array}$$
  Following this, the photoreceptor directed light displayed for an individual observer will be based on *A′* instead of the A-matrix (*A*). This arrangement ensures that the photoreceptor excitations for a given stimulus contrast remain constant across all individual observers.

### Confirmation of methods

**Timing: 30–60 min per participant per step**

At this stage in the preparatory steps for a silent substitution protocol, a confirmation should be performed to ensure that a true photoreceptor-directed stimulus is generated by the system. At least one of the following confirmations should be carried out, with the confirmation performed under similar viewing conditions to the experimental stimulus.

24. **Color matching:** Peripheral rod- and melanopsin-mediated color vision have a hue percept that can be matched by a signature combination of cone responses under conditions of photoreceptor isolation with silent substitution.^86^^,^^92^^,^^93^ This color match confirms that rod or melanopsin photoreceptor isolation has been achieved. The rod and melanopsin temporal color match are measured as follows:
  a. Temporally alternate a photoreceptor-directed reference light (i.e., rod or melanopsin) with cone-directed test lights that are independently adjustable along the L/(L+M), S/(L+M) and (L+M) directions. For the melanopsin directed lights, apply TWN during the inter-stimulus intervals to desensitize the penumbral cone intrusions.
  b. Have the observer adjust the cone excitations in the test light to make a perceptual match to the photoreceptor directed reference light. At the temporal color match the equivalent cone signals will be equal to:
    i. Mesopic rod percept: An increment in S/(L+M) and (L+M) and a decrement in L/(L+M).^92^
    ii. Photopic melanopsin percept: An increment in cone luminance (L + M) and a decrement in the S-cone excitation S/(L + M).^67^
25. **Critical Flicker Frequency (CFF):** The critical flicker frequency represents the maximum temporal resolution of a visual process.^94^ Isolation of a specific photoreceptor class (or classes) can be confirmed by comparing the CFF of the photoreceptor-directed stimulus to the known CFF for a photoreceptor class.
  a. Generate a cone-, rhodopsin-, and melanopsin-directed stimulus using the A′-matrix with the individual observer corrections. Apply TWN in the inter-stimulus intervals to desensitize the penumbral cone intrusions.
  b. Measure the critical flicker frequency for each photoreceptor-direction using the method of adjustment. Repeat the rod-directed measurement under scotopic and photopic illumination conditions to identify the intensity dependent CFF relationship.
  c. If an isolated photoreceptor-direction is achieved in the stimulus the measured CFF will have a maximum CFF:
    i. Cone-directed (LMS): above 60 Hz.
    ii. Rod-directed: approximately 30 Hz.^1^^,^^95^
    iii. Melanopsin-directed: approximately 5 Hz.^1^^,^^67^

***Note:*** The CFF is dependent on the stimulus size and contrast, viewing eccentricity and increases with illumination level.^96^ If there is some open-field error, the CFF for melanopsin and/or rhodopsin-directed stimuli will become closer to the cone CFF, with a diminishing difference with higher levels of intrusion.

26. **Bleach recovery test:** Following the exposure to an intense light that bleaches the photopigment, the visual response will slowly recover over time. Isolation of a designated photoreceptor class is confirmed by comparing the time taken for a photoreceptor-directed stimulus to become visible following offset of a bleaching light (i.e., the recovery time) to the known recovery times for the photopigment bleach levels induced by the intense light.
  a. Dark-adapt the participant for 15 min to eliminate effects of pre-light exposure.
  b. Generate separate cone-, rhodopsin-, and melanopsin-directed stimuli using the A′-matrix with the individual observer corrections. Apply TWN in the inter-stimulus intervals to desensitize the penumbral cone intrusions.
  c. Pre-expose one eye of the observer to a high intensity lamp (e.g., a 70 s, 120,000 Photopic Td/297,600 Scotopic Troland bleaching light). High intensity lamps should be optically filtered to eliminate any blue light hazard.
  d. Estimate the photopigment bleach levels using the framework developed by Hollins and Alpern^97^ and Thomas and Lamb^98^; this will determine the expected time course of recovery.^76^^,^^99^^,^^100^
  e. Immediately following offset of the bleaching light (time 0), periodically present the suprathreshold, photoreceptor-directed, pedestal stimuli to the eye that was bleached. During this post-bleach period (e.g., approximately 15-min, or until detected), record the times that the observer detects the photoreceptor-directed stimuli.
  f. If an isolated photoreceptor-direction is achieved, the time-course to recovery of the cone pathway will be faster than the rod pathway.^101^^,^^102^^,^^103^^,^^104^ The time course of recovery of the melanopsin pathway intermediate is between the rhodopsin and cone-opsins.^105^

**CRITICAL:** Photopigment bleaching can narrow the photoreceptor spectral nomograms^106^ and introduce time dependent inaccuracies in the estimated photoreceptor excitations applied in the A′-matrix.^105^ These artefacts decrease with increasing post-bleach durations. Any cone intrusion in the melanopsin- or rod-directed stimuli will reduce the time to see for an observer in the post-bleach period. However, because the cone contrast artefacts all have a lower contrast than the cone-directed pedestal stimuli the time to see will not be completely reduced to that of the cone-directed pedestal.

27. **Entopic percept of the retinal blood vessels:** Penumbral cone intrusions that are present in a melanopsin-directed stimulus will produce an entopic appearance of the retinal blood vessels (i.e., the Purkinje tree). The perception of retinal blood vessels can be utilized to test for the presence of penumbral cone intrusions in a melanopsin-directed stimulus:
  a. Present a melanopsin-directed stimulus to the observer to which the penumbral cones will be more sensitive to (e.g., a 10 Hz temporal grating stimulus).
  b. Task the observer with grading their perception of the Purkinje tree on a four-point grading (0 = No spatial structure; 1 = any spatial structure; 2 = faint Purkinje tree; 3 = strong Purkinje tree).^58^

## Key resources table

**Table:** 

REAGENT or RESOURCE	SOURCE	IDENTIFIER
**Deposited data**		

Toolbox for silent substitution	QUT Research Data Finder	https://doi.org/10.25912/RDF_1670844246774

**Other**		

ILT1700 Research Radiometer	International Light Technologies, Inc	https://www.intl-lighttech.com/products/ ilt1700-research-radiometer
EPP2000C-50μm Slit UV-VIS Spectrometer	StellarNet	https://www.stellarnet.us
SpectraScan PR655	Jadak	https://www.jadaktech.com/products/photo-research/spectrascan-pr-655/

## Step-by-step method details

The preliminary steps presented above are crucial to generating a truly photoreceptor-directed stimulus. The method of silent substitution can be applied under a wide range of experimental conditions^107^ once it has been confirmed that the generated stimulus is isolating the response of a single photoreceptor class. What follows in this section is an exemplar melanopsin-directed silent substitution experiment which puts together a selection of the preliminary steps discussed above and applies it to a melanopsin-directed temporal grating stimulus with temporal white noise in the inter-stimulus interval. A toolbox has been developed in MS-Excel to support this protocol and is available for download via the QUT Research Data Finder.^11^

### Pre-experiment preparation

**Timing: 1 day (excluding institutional approvals)**

1. **Institutional Approvals:** Perform experimental protocols in accordance with institutional research ethics approvals.
2. **Report participant demographics:** To facilitate replication of results, report the relevant factors from the criteria listed below in the methodological description of the experiment:
  a. State the recruitment method and inclusion and exclusion criteria for healthy and disease groups.
    ***Note:*** This may require an ophthalmic and/or medical examination with reference to the participant inclusion criteria. If applicable, include details of the randomisation to groups/treatments. Basic demographics can include age, information on the sex and specific relevant clinical features, for example presence/absence of ocular disease, systemic disease, sleep, mood disorders, genotypes or other disease or co-morbidity characteristics relevant for the research question/outcomes.
  b. Specify the clinical instruments (model and manufacturer) used for the participant classifications, for example, for visual acuity, contrast sensitivity, color vision, fundus photography, OCT, intraocular pressure, clinical grading scales, and documentation of medications (e.g., dosage, frequency).
  c. Determine the sample size needed based on the experimental design.
    ***Note:*** Power analyses can inform the minimum number of participants required for the statistical design. Many experiments using silent substitution include a small sample of participants and a high number of trial repeats. The effects of individual differences on the experimental outcomes can be examined by including a comprehensive classification and measurement of participant performance on as many dimensions as are feasible.

### Instrumentation

**Timing: Refer to before you begin timings for each step**

The instrumentation for this example is the 5-primary photostimulator introduced by Cao et al.^22^ The protocols apply to 5-primary displays designed to generate structured images:^17^

3. Choose a set of 5-primary lights as per steps 1–4 (Toolbox: *“Trimmed LED Spectra”*)^11^: [peak wavelengths (full widths at half maximum) at 456 nm (10 nm), 488 nm (11 nm), 540 nm (10 nm), 594 nm (14 nm), and 633 nm (15 nm)].
4. Calculate the 5 × 5 A-matrix using the corneal melanopsin, rhodopsin and CIE 10° standard observer L-, M-, and S-cone photoreceptor spectral sensitivities (Toolbox: *“Standard Observer (CIE)”*) and the measured 5 primary spectral outputs (Toolbox: *“A matrix”*).^11^

(Equation 15) $$\begin{array}{c} \vec{\beta }=\left[\begin{array}{ccccc} 2.04 & 27.0 & 0.04 & 0.00 & 0.00\\ 0.12 & 12.5 & 11.5 & 6.26 & 0.80\\ 0.11 & 7.04 & 10.2 & 8.25 & 8.81\\ 0.41 & 31.0 & 7.76 & 1.63 & 0.04\\ 0.53 & 39.3 & 4.31 & 0.45 & 0.01 \end{array}\right]\;\vec{p} \end{array}$$

5. Develop the system to present a uniform image to the observer. The photostimulator integrates the output of the 5 narrowband primary lights using optical fibers and a homogenizing bar and diffuser (e.g., Figure 2E). The uniform stimulus is imaged in the plane of a 2 mm artificial pupil in Maxwellian view (natural pupils) as a 30° outer diameter uniform field (10° diameter macular blocker).
6. Control the radiance of each primary independently using an LED driver, microcontroller and computer driving a pulse width modulation (PWM) frequency up to 488 Hz with 12-bit digitization per primary.
7. Conduct physical light calibrations in accordance with steps 11–16. This includes the 5 primary spectra (Toolbox: *“Trimmed LED Spectra”*), input-output linearization (Toolbox: *“*Linearization *samples”*) and modeling (Toolbox: *“*Linearization *Coefficients”*).^11^
8. Estimate the open field contrast errors as per step 17. We recorded that for a 17% melanopsin-directed incremental pulse set against a 2000 Td (4360 Scotopic Td) orange adapting field, the open field cone contrasts were L=0.0%,M=0.1%,S=1.3%,R=0.3%,&i=0.6%.
9. Estimate the penumbral cone contrast errors as per step 18. We recorded that for a 17% melanopsin-directed incremental pulse set against a 2000 Td (4360 Scotopic Td) orange adapting field (L=0.2%,M=0.5%,S=0.6%,&R=0.2%). In the cone isolating conditions, the rod contrast was always ≤0.3%.

**CRITICAL:** Eliminate blue-light hazards and/or any potential damaging effects of high illuminations that are greater than would be experienced in natural viewing conditions such as outdoors on the beach on a summer day, by suitable application of interference filters, neutral density filters, electrical and/or spectral control of the light output into the instrumentation.

### Individual observer calibrations

**Timing: 4 h per participant (HFP: 1 h, CFF:**3×1**hour per illumination level)**

10. Perform heterochromatic flicker photometry (HFP) as per step 21a). Scale the A matrix using the individual observer ratios. For our observer ${\vec{k}}_{p}=\left(p_{B}/p_{G},p_{C}/p_{G},1,p_{A}/p_{G},p_{R}/p_{G}\right)=\left(1.27,1.18,1.00,1.06,1.06\right)$ and the A′ matrix was derived (Toolbox: *“A matrix”*).^11^

(Equation 16) $$\begin{array}{c} \vec{\beta }=k_{p}\cdot A\vec{p}\\ \vec{\beta }=\left[\begin{array}{ccccc} 2.59 & 31.9 & 0.04 & 0.00 & 0.00\\ 0.15 & 14.8 & 11.5 & 6.62 & 0.84\\ 0.15 & 8.32 & 10.2 & 8.72 & 9.31\\ 0.52 & 36.7 & 7.76 & 1.72 & 0.04\\ 0.67 & 46.4 & 4.31 & 0.48 & 0.01 \end{array}\right]\vec{p} \end{array}$$

11. In addition to HFP, apply temporal white noise in the pre- and post-stimulus intervals (step 20) to supress penumbral cone errors (Toolbox: *“Stimulus”*).^11^
12. To confirm that the melanopsin-directed stimulus has truly isolated the melanopsin response, perform the critical flicker frequency test for each observer as per step 25. The CFF for a LMS=28%,R=15%&i=17% Michelson contrast stimulus was measured for each observer across a range of illuminations from 1 to 4.3 log scotopic Td. The melanopsin-directed stimuli remained invariant across illumination levels, indicating there was minimal rod (<3%) or cone intrusion.

### Experimental protocol

**Timing: 60 min per session**

#### Laboratory and participant preparation

13. Light and dark adaptation: The laboratory is darkened for the experiments. To eliminate the effects of pre-light exposure to indoor (artificial) or outdoor (daylight) illumination, each observer adapts in the dark-room for 15 min prior to commencement of the test sessions.

***Note:*** Experiments of rod mediated vision: Dark adapt for 30–40 min depending on the pre-light exposure conditions.^101^ Follow with a pre-adaptation to the background field before commencing the test protocol (e.g., 2 min for a 2000 Ph Td/4360 Sc Td adapting field).

The duration of pre- adaption is dependent on the viewing conditions and must be sufficiently long to allow maximal sensitivity for all photoreceptor pathways. It is good practice to conduct pilot experiments to determine the effect of dark-adaptation times on the outcome measures, and to optimize them to minimize the protocol duration.

**CRITICAL:** Any assumption that rods are saturated for the light levels should be verified with additional psychophysical, pupillography or electrophysiological measurements under the viewing conditions.

14. Report the laboratory measurement conditions including the room illumination (Lux), pre-dark or pre-light adaptation duration (or no adaptation) to the room illumination and/or background adaptation stimulus levels (scotopic, mesopic, photopic). When relevant, report the environmental conditions (time-of-day, temperature, humidity).
15. Observer tasks: It is important for participants to have a clear understanding of all experimental tasks, including the observer calibration. Visual and non-visual sensitivity can be affected by the measurement conditions of the laboratory; identify and control potential factors affecting internal and external validity. Effects of observer anticipation and habitation should be minimized. Randomize the order of presentation to limit the effects of fatigue and learning across multiple testing sessions. Ensure sufficient practice; exclude the data from the practice sessions.

#### Stimulus presentation

16. The stimulus characteristics and experimental paradigm will depend on the research question. For this example, we present a melanopsin-directed stimulus as a 1 s duration, 1 Hz temporal sinusoidal waveform (Toolbox: *“Stimulus”*)^11^ with temporal white noise presented in the inter-stimulus interval (2 s). The participant views the stimulus through the artificial pupil.
17. The mean adapting chromaticity is metameric to an equal energy white spectrum (SMLRi=[1,1/3,2/3,1,1]) (Toolbox: “*A matrix”*).^11^
18. At this point the system is functional for generating photoreceptor-directed stimuli. From here the experimental procedure and recorded data is dependent on the aims of the psychophysical, electrophysiology (including ERG and VEP) and pupillometry experiments.

## Expected outcomes

### Choice of primary lights

A set of primary lights chosen using this protocol can be optimized (step 3) to maximize the photoreceptor-directed contrast at any chromaticity or at a specified chromaticity. If the metric allowing any background adapting chromaticity is used, a chosen set of five primaries should be able to achieve Michelson contrasts of ≈ 40% for melanopsin-directed, and ≈30% for rhodopsin-directed stimuli (both at deep red chromaticities). If the metric for a specified background adapting chromaticity is used, then an equal energy white chromaticity should achieve Michelson contrasts of ≈20% for melanopsin-directed, and ≈18% for rhodopsin-directed stimuli.

These achievable contrasts are dependent on the available centre wavelengths and bandwidths of the considered primaries. Narrower bandwidth primaries increase the maximum photoreceptor contrast, but typically with lower output power. The chosen primaries should be evaluated as per step 4 to ensure there is sufficient bit resolution for each of the primary powers across the photoreceptor directed stimulus range.

### Display calibration

After calibration of the five-primary display (steps 11–16) the primary outputs of the system should be spatially aligned and homogenized (step 15), and the output frames temporally aligned (step 16). The range of spatial and temporal frequencies achievable by the system should be quantified. In addition, the spectral composition of the five-primaries should be well defined with the radiance output for each digital input known (step 14).

After these calibrations are complete the open-field and penumbral cone errors should be quantified as per steps 17–18, and displayed to a participant to ensure that any spectral errors in the system are imperceptible to a participant.

### Individual observer calibration

Physiological differences between individual observers can introduce differences between the calculated photoreceptor excitations and the actual photoreceptor excitations. The individual observer calibrations should account for the different L:M cone ratio and pre-receptoral filtering of each observer and the stimulus should be designed to supress penumbral cone intrusions. When the individual observer calibrations are performed, the confirmation of methods techniques (steps 24–27) should provide the expected results outlined in each step.

## Limitations

### Hardware capabilities

Silent substitution techniques are sensitive to inaccuracies arising from hardware limitations. This means that the system design will require sufficient bit depth resolution to accurately represent the stimulus. This should be evaluated by estimating the quantization error of the stimulus for a given photoreceptor-directed stimulus. Typically, systems with >8-bit resolution are required to measure threshold level vision. It should be noted that software corrections for linearization and spatial homogenization will compress the bit-depth that a system can produce and photoreceptor-directions with low single primary modulations will only modulate a reduced range.

In addition to the bit depth resolution of the hardware, the frame rate must be sufficient to not introduce temporal artefacts in temporally modulated stimuli. This can be mitigated by presenting stimuli with a windowed onset/offset and that avoids step changes in intensity.

### Systems with <5 primaries

Systems which do not have at least 5 spectrally independent primaries will not have control over the excitations of all five photoreceptor classes. This means that experiments which attempt to isolate a single class of photoreceptors will introduce uncontrolled excitation changes in another class of photoreceptor. Specifically, because the rhodopsin and melanopsin spectral responses are correlated, a stimulus with a maximal change in melanopsin excitation will introduce a maximal change in rhodopsin excitation as a confounding factor. An example method for studying melanopsin, without controlling rhodopsin, is described elsewhere.^108^

For a standard display device with 3 primaries, the methods described here could be used to study photopic cone-mediated vision (albeit with uncontrolled melanopsin and rhodopsin excitation). With 4-primaries, one photoreceptor class is left uncontrolled (e.g., rhodopsin or melanopsin). This can be done by removing the A-matrix rows that relate to the untargeted photoreceptors (Equation 2).

### Systems with >5 primaries

A system can be designed with more than five primaries to achieve ancillary benefits, including an increased gamut of background adapting chromaticities which can achieve a higher photoreceptor-directed contrast. Only five primaries are needed to achieve the maximum photoreceptor-directed contrast for a single photoreceptor class, but more than five primaries could also be used to target the maximum contrast of different photoreceptor classes.

### Observer calibrations

The requirement of the application of individual observer calibrations to isolate the melanopsin response can be an onerous and time-consuming experimental procedure. This often limits the number of observers which can participate in a study and can be challenging for participants with ophthalmic, systemic, or neurodegenerative diseases. This is often overcome in silent substitution protocols by increasing the number of repeats on a smaller sample of observers, where individual observer calibrations are only needed once per observer. There are opportunities to simplify the individual observer calibration procedures to promote their application in larger sample size studies.

### Melanopsin bistability and tristability

The photochemistry of melanopsin suggests that melanopsin can exist in one signaling and two electrically silent states.^109^^,^^110^ This property of melanopsin has been used to suggest that the electrically silent states may play a role in pigment regeneration.^111^ *In vivo* studies on human participants have not detected evidence of the potentiating effect of long wavelength light on melanopsin function.^112^^,^^113^

## Troubleshooting

### Problem 1

Instability in the spectral and/or radiance of the primary lights (step 14).

### Potential solution

The output characteristics of an LED vary with the temperature of the LED (step 14). This can be seen as spectral drift in measurements of the primary outputs or as changing radiance outputs (i.e., reducing) after onset of the LED. This can be mitigated by:

• Applying appropriately sized heat sinks to each of the primary LEDs. If passive heatsinking is insufficient, then a cooling fan should be considered.
• Spectral filtering of the primaries can limit spectral drift of the primaries if the spectral content of each primary is mostly determined by the shape of the spectral filter. However, this method alone will still result in the radiance output of the LEDs changing as the temperature of the LEDs fluctuate.

It should be noted that modulating a primary with a DMD instead of LED PWM can avoid dynamic changes in LED temperatures. In this case, an LED is driven with a 100% duty cycle at a stable current for the available primary heatsinks, meaning the LED will reach a stable equilibrium after the device is switched on and the DMD modulation will be independent of the LED temperature.

### Problem 2

The number of unique levels from the photoreceptor-low to photoreceptor-high light condition is insufficient for some primaries (step 4).

### Potential solution

With a five primary device there is only a single modulation direction that will silently modulate a chosen photoreceptor. As an example, a melanopsin-directed stimulus may require a large change in the red primary whilst requiring very little change in the violet primary. The primary which changes over a larger proportion of its dynamic range will have more unique levels between the melanopsin-high and melanopsin-low condition and so the quantization error over a waveform will be larger for the primary which covers a lower dynamic range.

To combat this effect, individual primaries can have their output increased (e.g., by adding more LEDs, or increasing the current through the LEDs) or decreased (e.g., by attenuating the primary output level with a neutral density filter or reducing current) to ensure the photoreceptor-directed stimuli covers a larger and more even proportion of the primaries dynamic range. Alternatively, if interchanging primaries is achievable in the display, then choosing a set of five primaries which more evenly changes the power across primaries over the stimulus conditions could be preferable.

### Problem 3

The instrumentation for stimulus generation has inconsistent frame time or frame rate (step 16).

### Potential solution

When generating five primary stimuli with a display which takes inputs from multiple computers or graphics outputs there is a risk that the two video streams are temporally misaligned or are independently dropping video frames. Some potential solutions to this issue include:

• Upgrading the graphics processing unit (GPU) and central processing unit (CPU) on the controlling computers to avoid dropping video frames given the number of graphics outputs, screen resolution and frame rate.
• Avoid, where possible, real-time processing of the video frame outputs during time critical portions of the experimental protocol.
• Moving to a software package/language with lower level control of system interrupts (step 9).

## Resource availability

### Lead contact

Further information and requests for resources should be directed to and will be fulfilled by the lead contact, Andrew J. Zele (andrew.zele@qut.edu.au).

### Materials availability

This study did not generate new unique reagents.

## Data Availability

The Toolbox is available open access through the QUT Research Data Finder.^11^
